# Penetrating lung injury during Nuss procedure for pectus excavatum

**DOI:** 10.1186/s13019-020-01236-6

**Published:** 2020-07-23

**Authors:** Do Yeon Kim, Jin Yong Jeong

**Affiliations:** Department of Thoracic and Cardiovascular Surgery, Incheon St. Mary’s Hospital, College of Medicine, The Catholic University of Korea, 56 Dongsu-ro, Bupyeong-gu, Incheon, 21431 Republic of Korea

**Keywords:** Pectus excavatum, Nuss procedure, Complication, Thoracoscopy

## Abstract

Life-threatening complications including cardiac perforation by the clamp or pectus bar during Nuss procedure have rarely been occurred. A rare case of lung entrapment between the pectus bar and chest wall after Nuss procedure was also reported. Thoracoscopy allows for direct visualization of the operative field, which prevents or promptly perceive these intrathoracic organ injuries. Recently, we encountered a case of penetrating lung injury during the Nuss procedure for pectus excavatum. And we agree with Mennie et al. who concluded thoracoscopic vision during Nuss procedure reduces the risk of major complication. In addition, we would like to emphasize to keep in mind what to check for routines with thoracoscopy during Nuss procedure.

Correspondence

Dear Sir,

We read with great interest the article by Mennie et al. [1], in which the authors retrospectively reviewed 217 patients with pectus excavatum to prove whether the operative complications would be reduced by using thoracoscopy. The authors did not initially use thoracoscope for 122 Nuss procedures, but they have used one for 95 since 2009. And they found that thoracoscopic assistance during Nuss procedure reduces the risk of major complication.

Life-threatening complications including cardiac perforation by the clamp or pectus bar during Nuss procedure have rarely been occurred [2, 3]. Moss et al. reported a case of cardiac perforation with the clamp passing through the right atrium and the right ventricle. They promptly made a midline sternotomy, initiated cardiopulmonary bypass and repaired the cardiac injury [2]. Gips et al. reported a case of cardiac perforation with the pectus bar penetrating the anterior aspect of the heart, leading to death [3]. Bilgi et al. reported 15 cases of lung parenchymal laceration, which occurred during the blind insertion of the trocar or the dividing lung adhesions in the Nuss procedure [4]. Kim et al. reported a case of lung entrapment between the pectus bar and chest wall after Nuss procedure [5]. They found the entrapment of right middle lobe when performing thoracoscopic surgery to treat pneumothorax with the persistent collapsed lung which developed on postoperative fourth day. They thought that the damaged lung might be related with air leak. Henry et al. reported a case of lung laceration from adhesions between the pleura and the pectus bar, which occurred during the removal of the bar and required lobectomy to the control bleeding [6].

Recently, we encountered a case of penetrating lung injury caused by pectus bar, a rare complication, confirmed by thoracoscopy. A female patient underwent Nuss procedure under assistance of 2-mm needlescope during dissecting substernal space. After fixing the pectus bars, we found lung injury penetrated by the pectus bar with using needlescope and repositioned the bar to avoid postoperative complications including air leak and bleeding (Fig. 1). Pneumothorax was occurred postoperatively and resolved after 5 days, and the patient was discharged to the normal course.

**Fig. 1:**
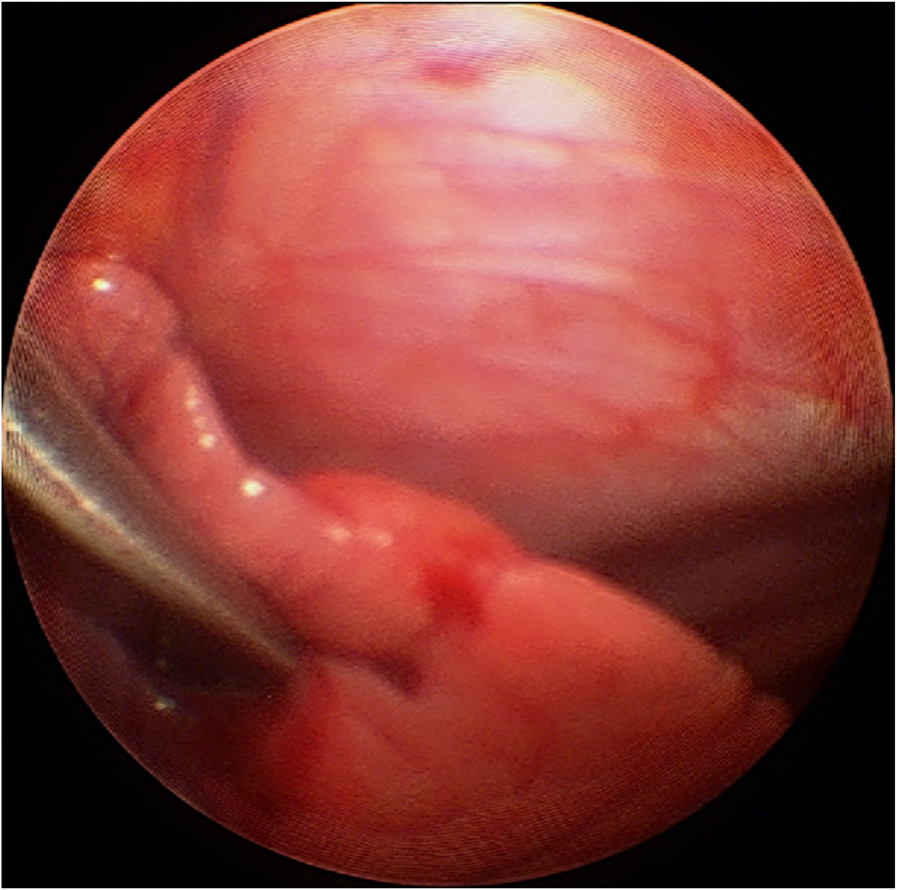
Thoracoscopy during Nuss procedure, showing the pectus bar penetrating the upper lobe of left lung

Thoracoscopy allows for direct visualization of the operative field, which prevents or promptly perceive the intrathoracic organ injury. We have performed the Nuss procedure under thoracoscopic assistance. In this case, after dissecting the substernal space and inserting the introducer through the space under the needlescope, the needlescope was removed to facilitate the manipulation of the introducer while determining the exit of the opposite chest wall. Perhaps this is when the penetrating lung injury occurred. To avoid this complication, a thoracoscopy should be performed until the introducer penetrates the opposite chest wall.

We would like to share our experience of using the thoracoscopy as a routine in Nuss procedure and emphasize the importance of the use of the thoracoscopy. We routinely check the lowest point of chest wall depression with the thoracoscopy at the beginning of the procedure to determine where the pectus bar is placed. Next, make sure to avoid heart damage when dissecting the substernal space and to prevent intrathoracic organ injury including lung perforation, which may occur during the introducer insertion, as in this case. After inserting the pectus bar, check whether the pectus bar is well positioned and check for complications such as heart damage or bleeding. In addition, we checked for lung injury including laceration and entrapment due to the rotation of the pectus bar.

In summary, we encountered a case of penetrating lung injury during the Nuss procedure in the pectus excavatum patient. We agree to that thoracoscopic vision during Nuss procedure reduces the risk of major complication. In addition, we would like to emphasize to keep in mind what to check for routines with thoracoscopy during Nuss procedure.

## Data Availability

Not applicable.
